# Ferroptosis and gastric cancer: from molecular mechanisms to clinical implications

**DOI:** 10.3389/fimmu.2025.1581928

**Published:** 2025-08-11

**Authors:** Hongyu Zhao, Limei Ao, Sorina, Yuxia Wei, Hong Zhen Yin, Nan Zhang, Xiao Qing Lee, Feng Li Du, Gai Lan Zhou

**Affiliations:** ^1^ Department of Gastroenterology, The Traditional Chinese and Mongolian Medicine Hospital of Hohhot, Huhhot, China; ^2^ College of Traditional Chinese Medicine, Inner Mongolia Medical University, Huhhot, China; ^3^ Department of Gastroenterology, Inner Mongolia Autonomous Region Traditional Chinese Medicine Hospital, Huhhot, China; ^4^ Oncology Center, Inner Mongolia Autonomous Region Traditional Chinese Medicine Hospital, Huhhot, China; ^5^ College of Traditional Chinese Medicine, Baotou Medical University, Baotou, China

**Keywords:** gastric cancer, ferroptosis, lipid peroxidation, GPx4, treatment resistance

## Abstract

Gastric cancer, one of the leading causes of cancer-related mortality globally, faces challenges in treatment due to limitations in surgery, chemotherapy resistance, and high recurrence rates. Ferroptosis, an iron-dependent form of cell death, induces cell membrane rupture through dysregulated iron metabolism, lipid peroxidation, and the accumulation of reactive oxygen species (ROS), offering a promising therapeutic avenue for gastric cancer treatment. This article systematically explores the core mechanisms of ferroptosis, including iron overload catalyzing lipid peroxidation via the Fenton reaction, dysregulation of antioxidant systems (such as GPX4 and FSP1), and their associations with gastric cancer cell proliferation, metastasis, and resistance. Studies indicate that abnormalities in iron metabolism in gastric cancer cells, such as upregulation of TFR1 and dysregulated ferritin storage, significantly promote ferroptosis sensitivity, while ferroptosis inducers (such as Erastin and RSL3) can enhance chemotherapy sensitivity and reverse resistance by inhibiting GPX4 or system Xc-. Preclinical experiments confirm that targeting ferroptosis-related pathways (such as the USP7/SCD axis and ABCC2-mediated glutathione efflux) effectively inhibits tumor growth and metastasis. However, the dual-edged effect of ferroptosis warrants caution regarding its oxidative damage risk to normal tissues and potential pro-metastatic mechanisms. This article further proposes the potential of ferroptosis biomarkers (such as 4-HNE and GPX4) in early diagnosis and prognosis assessment of gastric cancer and emphasizes the need for precision medicine to optimize ferroptosis-targeted strategies, balancing efficacy and safety. Ferroptosis opens a new avenue for gastric cancer treatment, but its clinical translation still requires in-depth mechanistic exploration and personalized treatment plan design.

## 1 Introduction

Gastric cancer(GC) is one of the leading causes of cancer-related mortality worldwide, particularly in East Asia, where its incidence and mortality rates are significantly high, making it a major cause of cancer-related deaths in the region. According to data from the International Agency for Research on Cancer (IARC), GC causes approximately 780,000 deaths globally each year, ranking among the leading causes of cancer mortality worldwide (1). The high incidence of GC is strongly associated with multiple factors, including unhealthy dietary patterns, *Helicobacter pylori*(HP) infection, genetic predisposition, and environmental exposures (2). Current treatment strategies for GC primarily depend on conventional modalities, including surgery, chemotherapy, and radiotherapy. Although these approaches can modestly improve patient survival, they are still associated with numerous limitations and challenges. Surgical intervention is typically limited to early-stage GC and is often associated with a high rate of postoperative recurrence. Although chemotherapy and radiotherapy can help control tumor progression in certain patients, these therapies are frequently accompanied by severe adverse effects, such as bone marrow suppression, immunosuppression, and gastrointestinal dysfunction (3). In addition, drug resistance remains a major obstacle in the treatment of GC, particularly in advanced-stage patients, where it significantly increases the complexity of therapeutic management (4). Therefore, the identification of novel therapeutic targets and the development of innovative treatment strategies have become critical priorities in contemporary GC research.

In recent years, ferroptosis, an emerging iron-dependent form of cell death, has attracted growing interest among researchers. Unlike traditional forms of cell death, such as apoptosis, the hallmark of ferroptosis is the excessive accumulation of iron, which triggers lipid peroxidation, ultimately leading to the rupture of the cell membrane and cell death (5). Ferroptosis plays a pivotal role not only in neurodegenerative diseases but also in the initiation and progression of various cancers (6). In GC, dysregulated iron metabolism and iron overload are particularly prominent, providing a potential basis for the investigation of ferroptosis. Excessive iron accumulation may promote lipid peroxidation, thereby contributing significantly to the proliferation, metastasis, and therapeutic resistance of GC cells (7). By modulating iron metabolism or inducing ferroptosis, it is anticipated that the limitations of traditional therapeutic approaches can be overcome, particularly in addressing chemotherapy and radiotherapy resistance. Ferroptosis inducers, as a potential cancer treatment strategy, have demonstrated efficacy in the treatment of various tumors (8). This article aims to explore the therapeutic potential of ferroptosis in GC, with a particular emphasis on recent advances in its role as an emerging treatment target. This review will provide a comprehensive overview of the mechanisms underlying ferroptosis, the dysregulation of iron metabolism in GC, and the roles of ferroptosis in tumor cell proliferation, metastasis, and immune evasion. In addition, we will summarize recent preclinical advances in the use of ferroptosis inducers for GC treatment and explore their potential future applications, aiming to offer new perspectives for therapeutic strategies against GC.

## 2 Mechanisms of ferroptosis

### 2.1 Iron metabolism imbalance

Iron is an essential intracellular element involved in various critical biochemical processes, including oxygen transport, DNA synthesis, and cellular respiration. Under physiological conditions, iron homeostasis is tightly regulated by key molecules such as transferrin (Tf), transferrin receptor (TfR), and ferritin, thereby maintaining systemic iron balance. T Transferrin is responsible for transporting iron through the bloodstream and delivering it to cells, while transferrin receptors mediate cellular iron uptake via receptor-mediated endocytosis. Ferritin functions as an intracellular iron-storage protein, sequestering excess iron to prevent iron overload and the associated oxidative damage. However, when iron intake exceeds physiological demand or storage mechanisms become dysregulated, the resulting iron accumulation creates a cellular environment conducive to ferroptosis. One of the key aspects of iron metabolism imbalance is the excessive accumulation of iron, particularly the buildup of free iron. Free iron primarily exists in the form of Fe²^+^ (ferrous ion), which can catalyze the production of highly reactive hydroxyl radicals through the Fenton reaction with hydrogen peroxide (H_2_O_2_). These radicals are highly reactive and capable of attacking cellular lipids, proteins, and DNA, causing severe structural damage and ultimately leading to cell death (9). Excess iron not only directly generates reactive oxygen species (ROS) through the Fenton reaction but also activates multiple oxidative stress pathways, thereby amplifying ROS production. The excessive accumulation of ROS induces lipid peroxidation of cellular membranes and disrupts the structure and function of other critical biomolecules, ultimately accelerating the process of cell death (10). Therefore, dysregulation of iron metabolism directly initiates oxidative stress responses, and the sustained accumulation of ROS ultimately accelerates the progression of ferroptosis.

### 2.2 Lipid peroxidation system

Lipid peroxidation is a central mechanism underlying ferroptosis and plays a critical role in its progression. Hydroxyl radicals, primarily generated through the Fenton reaction, serve as key initiators of this process. These reactive species interact with polyunsaturated fatty acids (PUFAs) in cellular membranes, thereby initiating and propagating lipid peroxidation. The cell membrane is primarily composed of a phospholipid bilayer, which contains a high concentration of polyunsaturated fatty acids, making the membrane particularly susceptible to oxidative damage (5). Lipid peroxidation products, including lipid hydroperoxides (LOOH), 4-hydroxy-2-nonenal (4-HNE), and malondialdehyde (MDA), are highly cytotoxic molecules. LOOH, as a key intermediate in the lipid peroxidation cascade, can further react with cellular membrane components, thereby exacerbating membrane damage and contributing to ferroptotic cell death (11). 4-HNE is a major lipid peroxidation product with strong cytotoxic properties. It can form covalent adducts with proteins and DNA, generating reactive intermediates that contribute to cellular dysfunction, senescence, and death (12). MDA is also a hallmark product of lipid peroxidation, known for its strong mutagenic properties. It can react with intracellular nucleic acids, forming crosslinks that accelerate cell death. The occurrence of lipid peroxidation directly disrupts the structure and function of the cell membrane. The phospholipid bilayer of the cell membrane serves as a barrier to maintain the balance of substances between the intracellular and extracellular environments. Any damage to the membrane triggers changes in membrane permeability, leading to ion imbalance, cell lysis, and loss of function. In ferroptosis, lipid peroxidation products enhance the risk of membrane rupture by altering the membrane’s fluidity and stability, ultimately resulting in cell death (13).

Lipid peroxidation not only disrupts the structural integrity of cellular membranes but also induces covalent crosslinking with intracellular proteins, RNA, and DNA, ultimately resulting in the loss of cellular function. Studies have demonstrated that lipid peroxidation products can aggravate oxidative stress and promote cell death by activating various intracellular receptors, enzymes, and signaling pathways (14). Emerging evidence suggests a strong interplay between lipid peroxidation products and other forms of regulated cell death. For example, products such as 4-HNE and MDA not only cause direct membrane damage but also modulate intracellular signaling pathways, activating programmed cell death processes such as apoptosis, thereby facilitating the progression of ferroptosis (15). Overall, lipid peroxidation constitutes a central event in the execution of ferroptosis. The accumulation of lipid peroxidation products compromises membrane integrity, activates multiple cell death signaling pathways, and ultimately results in irreversible cellular damage.

### 2.3 Antioxidant pathways and ferroptosis

Ferroptosis is an iron-dependent, non-apoptotic form of cell death, typically accompanied by a significant increase in intracellular ROS levels. The accumulation of ROS triggers lipid peroxidation within the cell, leading to cell death. However, intracellular antioxidant mechanisms can effectively inhibit the occurrence of ferroptosis by scavenging these ROS. Glutathione peroxidase 4 (GPX4) is one of the most critical antioxidant enzymes in ferroptosis. It prevents the propagation of lipid peroxidation by catalyzing the reduction of lipid peroxides in conjunction with glutathione (GSH). The activity of GPX4 is a key factor in inhibiting ferroptosis; a deficiency in GPX4 or inhibition of its activity leads to the accumulation of lipid peroxides, further exacerbating ferroptosis. Enhancing GPX4 activity has become an important strategy for mitigating the progression of ferroptosis and treating ferroptosis-related diseases (16). In addition to GPX4, ferroptosis suppressor protein 1 (FSP1) is another important antioxidant factor. Unlike the glutathione system, FSP1 directly reduces lipid peroxides, preventing their accumulation and thereby inhibiting ferroptosis (17). The mechanism of action of FSP1 involves the reduction of lipid peroxides in the cell membrane, preventing their propagation and subsequent membrane damage. Particularly in cases where GPX4 is inactivated, FSP1 provides an independent alternative mechanism to suppress ferroptosis (18). Studies have shown that FSP1 expression is closely associated with cellular resistance to iron overload. Upregulation of FSP1 significantly delays the onset of ferroptosis (19). The identification of FSP1 has introduced a novel perspective on the regulation of ferroptosis. Similar to GPX4, FSP1 is a critical component of the cellular antioxidant defense system. Recent studies have focused on activating FSP1 function through small-molecule compounds as a promising therapeutic strategy for ferroptosis, particularly in cells with compromised GPX4 activity. Ferritin also plays a crucial role in the antioxidant system. It primarily prevents free iron from participating in the Fenton reaction by storing excess intracellular iron. Studies have shown that the overexpression of ferritin significantly delays the onset of ferroptosis. By regulating ferritin synthesis, cells can restrict the accumulation of free iron, alleviate oxidative stress, and inhibit the onset of ferroptosis (20).

**Figure 1:**
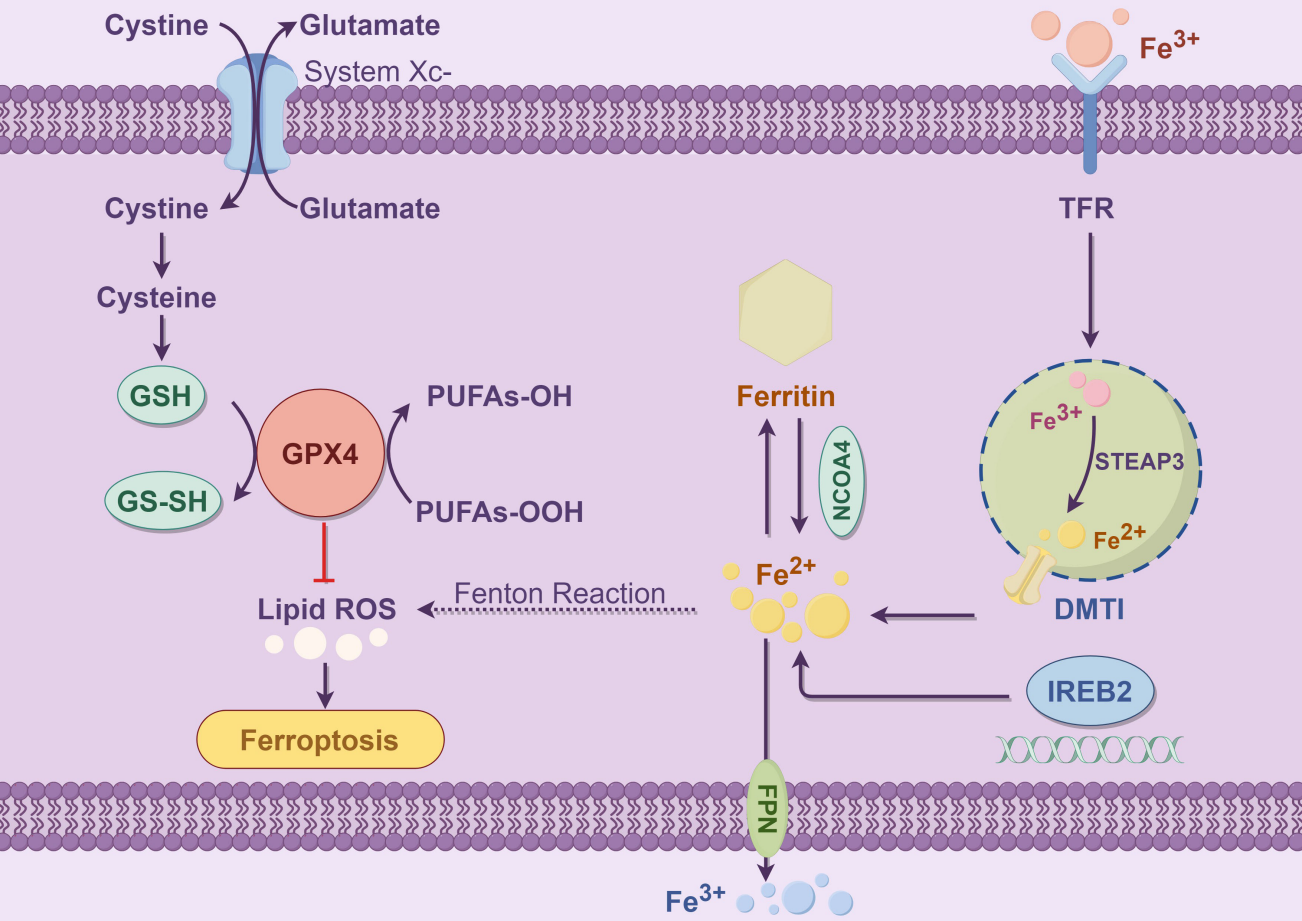
This figure illustrates the core molecular mechanisms of ferroptosis, with a particular emphasis on the interactions among iron metabolism, glutathione metabolism, and lipid peroxidation. Ferroptosis is an iron-dependent form of programmed cell death, primarily characterized by lipid peroxidation of polyunsaturated fatty acids (PUFAs) within the cell membrane, ultimately leading to cell death. The left panel depicts the antioxidant defense mechanism: Cells utilize System Xc^-^ (a cystine/glutamate antiporter) to export extracellular glutamate while simultaneously importing cystine. System Xc^-^ is a heterodimer composed of SLC7A11 and SLC3A2, where SLC7A11 serves as the functional subunit mediating cystine uptake (23).The imported cystine is subsequently reduced to cysteine, which serves as a precursor for glutathione (GSH) synthesis catalyzed by glutathione synthetase. GSH, in cooperation with glutathione peroxidase 4 (GPX4), eliminates lipid hydroperoxides (PUFAs-OOH), thereby preventing the excessive accumulation of lipid reactive oxygen species (ROS) and inhibiting the onset of ferroptosis (24). GPX4 is the core enzyme responsible for reducing lipid hydroperoxides into non-toxic PUFAs-OH, and its inactivation directly triggers ferroptosis (25).The right panel illustrates the iron metabolism pathway: Iron uptake is mediated by transferrin receptor (TFR), which facilitates the internalization of Fe³^+^. Intracellular six-transmembrane epithelial antigen of prostate 3 (STEAP3) then reduces Fe³^+^ to Fe²^+^. Subsequently, divalent metal transporter 1 (DMT1) transports Fe²^+^ into the cytoplasm (26). Free ferrous iron (Fe²^+^) can participate in the Fenton reaction, generating hydroxyl radicals (•OH), which initiate and propagate lipid peroxidation chain reactions, thereby acting as one of the key triggers of ferroptosis (27). Cells store excess Fe²^+^ in ferritin, a cytosolic iron storage protein. Nuclear receptor coactivator 4 (NCOA4) mediates the selective autophagic degradation of ferritin, a process known as ferritinophagy, which releases Fe²^+^ and exacerbates intracellular iron overload (28). Iron-responsive element-binding protein 2 (IREB2) regulates the expression of iron metabolism-related genes, such as TFR, DMT1, and ferroportin (FPN). This regulation contributes to the maintenance of intracellular iron homeostasis at the systemic level. Meanwhile, FPN exports Fe²^+^ out of the cell, thereby preventing iron overload (29, 30). In summary, disruptions in iron uptake, storage, transport, and the antioxidant system can lead to the excessive accumulation of polyunsaturated fatty acid (PUFA) peroxides and elevated levels of lipid ROS, thereby initiating the process of ferroptosis.

In addition to GPX4, FSP1, and ferritin, other antioxidant enzymes, such as superoxide dismutase (SOD) and NADPH oxidase (NOX), also contribute to the suppression of ferroptosis. NOX and SOD mitigate oxidative damage induced by iron overload by scavenging intracellular ROS, thereby inhibiting the onset of ferroptosis (21, 22). Especially under conditions of iron overload, antioxidant enzymes such as NOX and SOD help maintain cellular stability by regulating the redox balance. With the increasing understanding of the mechanisms of ferroptosis, enhancing the function of the cellular antioxidant system, particularly by boosting GPX4 activity and FSP1 expression, has become a key strategy for preventing ferroptosis. Many small molecule drugs are currently under development, aiming to regulate these antioxidant pathways and, consequently, control the occurrence of ferroptosis. The following figure illustrates the basic molecular mechanism of ferroptosis (**Figure 1**).

## 3 The pervasive role of ferroptosis in tumor diseases

### 3.1 Ferroptosis and cancer cell proliferation

Ferroptosis has been recognized as a pivotal process in the pathogenesis and therapeutic response of various cancers. Accumulation of excess iron has been shown to activate lipid peroxidation pathways, with the generated peroxides compromising the integrity of the cell membrane, thereby impeding tumor cell proliferation (31). This mechanism is intricately linked to the rapid proliferation and elevated metabolic demands of cancer cells.

In various types of cancer, ferroptosis induces cell cycle disruption through redox imbalance, thereby affecting cellular proliferation. Particularly under conditions of iron overload, the accumulation of ROS not only damages the cell membrane structure but also activates key signaling molecules within the cell cycle, leading to the inhibition of cell proliferation. For instance, in colon cancer cells, iron overload impairs cell membrane integrity through oxidative stress, consequently inhibiting the proliferation and survival of cancer cells (32). This process not only promotes the accumulation of lipid peroxides but also triggers apoptotic pathways, leading to tumor cell death. Furthermore, ferroptosis further regulates cell proliferation by influencing different stages of the cell cycle. For instance, during the G2/M phase, ferroptosis induces cell cycle arrest through the generation of ROS, thereby preventing the proliferation of cancer cells (33). By inhibiting the progression of the cell cycle, ferroptosis suppresses the proliferation of cancer cells, thus emerging as a potential therapeutic strategy.

### 3.2 Ferroptosis and cancer cell metastasis

Studies have shown that ferroptosis not only affects tumor cell proliferation but also regulates the metastasis of cancer cells by modulating various components of the tumor microenvironment (34). Ferroptosis disrupts the cell membrane, thereby affecting the migration and invasion of cancer cells. This membrane damage not only alters cell morphology but also disrupts the structure and function of the cytoskeleton, thereby inhibiting the motility and metastatic potential of cancer cells. Studies have shown that in breast cancer, ferroptosis can also modulate the polarization of macrophages within the tumor microenvironment, suppressing the function of M2 macrophages and consequently reducing cancer cell migration and invasion (35). In this process, ferroptosis alters the redox status of the tumor microenvironment through the generation of ROS, further influencing the interaction between cancer cells and immune cells. Additionally, ferroptosis affects the metastatic process through multiple pathways, including the remodeling of the extracellular matrix, stabilization of the cytoskeleton, and regulation of angiogenesis (34). Studies have found that ferroptosis inhibits angiogenesis in breast cancer cells by regulating metastasis-related factors, thereby reducing tumor metastasis (36). Thus, ferroptosis can significantly alter the metastatic potential of cancer cells by affecting processes such as cytoskeletal remodeling and angiogenesis, offering new perspectives for cancer therapy.

### 3.3 Tumor microenvironment and immune evasion

Iron metabolism within the tumor microenvironment (TME) is one of the key factors influencing tumor growth, metastasis, and immune evasion. Dysregulation of iron metabolism may promote oxidative stress and induce ferroptosis, further affecting the behavior of tumor cells (37). The sources of iron within the TME are diverse, including iron from the serum, tumor-associated macrophages (TAMs), and the endogenous iron regulatory mechanisms of tumor cells. In tumor cells, iron accumulation and iron endocytosis are regulated by TFR and associated ferritin, processes which can trigger cell death through the mechanism of ferroptosis or promote cancer progression (38). The accumulation of iron creates a favorable environment for tumor cells during immune evasion, playing a significant role in tumor progression (39). In TME, ferroptosis activates the NLRP3 inflammasome and cytokine release, thereby enhancing the inflammatory response and promoting immune evasion (40). Induction of ferroptosis impairs T cell function, contributing to immune evasion by tumor cells. In particular, TAMs promote iron release into the TME by upregulating FPN expression under the influence of the hypoxia-inducible factor 1-alpha (HIF-1α) and interleukin-6 (IL-6) signaling axis. HIF-1α activation in hypoxic regions induces IL-6 secretion, which in turn stimulates FPN expression in macrophages, enhancing iron export and availability to tumor cells (41). This HIF-1α/IL-6/FPN pathway reshapes the iron distribution within the TME, favoring tumor cell proliferation and impairing anti-tumor immune responses. Furthermore, ferroptosis may regulate the iron storage proteins in the tumor microenvironment, altering the iron demand of immune cells and thereby influencing the tumor’s immune tolerance (38). Additionally, the impact of ferroptosis on tumor immune evasion is also mediated through its effect on the presentation of tumor cell surface antigens and the expression of immune checkpoint molecules. By modulating ferroptosis, it may be possible to restore the immune system’s ability to recognize and attack tumor cells (42). Therefore, the interplay between ferroptosis and tumor immune evasion may offer valuable insights for the development of innovative immunotherapeutic strategies.

### 3.4 Ferroptosis and antitumor therapy

Chemotherapy is one of the standard approaches for cancer treatment; however, tumor cells often develop resistance, which diminishes its therapeutic efficacy. Recent studies have indicated that ferroptosis, an emerging iron-dependent cell death mechanism, may play a pivotal role in overcoming chemotherapy resistance. By inducing oxidative stress and lipid peroxidation, ferroptosis can trigger cell death in chemotherapy-resistant tumor cells, potentially improving the outcomes of conventional therapies (43). Ferroptosis, by promoting lipid peroxidation and the accumulation of iron ions, can significantly enhance the sensitivity of tumor cells to certain chemotherapeutic agents. This mechanism suggests that ferroptosis induction may be a potential strategy to overcome drug resistance and improve the therapeutic efficacy of chemotherapy in cancer treatment (44). In some experimental models, the combination of chemotherapeutic agents and ferroptosis inducers has significantly enhanced anti-tumor efficacy. For example, studies have shown that the use of ferroptosis inducers such as Erastin in combination with chemotherapy drugs notably improves therapeutic outcomes against various types of tumors, particularly those that exhibit resistance to traditional chemotherapy (45, 46). Therefore, exploring the use of ferroptosis as an adjunctive therapy in combination with chemotherapy holds significant clinical potential for overcoming drug resistance and enhancing therapeutic efficacy.

Immunotherapy has emerged as a major breakthrough in cancer treatment in recent years, particularly with the use of immune checkpoint inhibitors (ICIs). However, the therapeutic efficacy of immunotherapy is not always significant across all patients, with tumor immune escape mechanisms being a key contributing factor. The relationship between ferroptosis and immunotherapy has also gained increasing attention. Studies suggest that ferroptosis can influence the activity of immune cells in TME, thereby affecting the outcomes of immunotherapy (47). On the other hand, the combination of immune checkpoint inhibitors (ICIs), such as PD-1/PD-L1 inhibitors, with ferroptosis inducers has been shown to significantly enhance therapeutic efficacy, particularly in ferroptosis-sensitive tumor cells (46). The combination of ferroptosis and immunotherapy may become an important direction in future cancer treatment, particularly in overcoming resistance to immunotherapy and enhancing therapeutic efficacy. The table below presents the currently well-researched ferroptosis inducers and their target mechanisms (**Table 1**).

**Table 1:** Common ferroptosis inducers and their mechanisms.

Ferroptosis inducers	Targets	Mechanism	Clinical Development Status	References
Erastin	System Xc-	Inhibition of System Xc-, depletion of glutathione, and increase of ROS.	Preclinical;mechanism extensively studied	(15, 48)
RSL3	GPX4	Inhibition of GPX4, reducing antioxidant activity.	Preclinical; demonstrated efficacy in multiple models	(49)
Lip-1	ACSL4	Inhibition of ACSL4, reducing fatty acid metabolism, and promoting ferroptosis.	Preclinical; primarily basic research	(50)
Artemisinin	System Xc-	Inhibition of System Xc-, depleting glutathione, and increasing ROS.	Preclinical; studied across multiple cancer types	(51)
Shikonin	NADPH oxidase	Inhibition of NADPH oxidase, enhancing ROS generation.	Preclinical; initial mechanistic support	(52)
Sodium Selenite	NADPH oxidase	Inhibition of NADPH oxidase activity, thereby increasing intracellular ROS production.	Preclinical; nutrition-related research	(53)
FIN56	GPX4、SQS	Induces GPX4 degradation and activates the SQS pathway to promote lipid peroxidation.	Preclinical; confirmed in animal studies	(54)
DHPO	USP7/SCD	USP7 inhibition destabilizes SCD, promoting lipid ROS accumulation.	Preclinical; mainly focused on target validation	(55)
FINO2	Fe²^+^-induced oxidative stress	Promotes ROS production via Fe²^+^ oxidation, indirectly inactivates GPX4, and induces lipid peroxidation.	Preclinical; efficacy demonstrated in animal studies	(56)
ML-162	GPX4	High-affinity GPX4 inhibition rapidly induces lipid ROS accumulation.	Preclinical; well-defined target specificity	(57)

## 4 Ferroptosis and GC

### 4.1 Molecular mechanisms of GC

**Figure 2:**
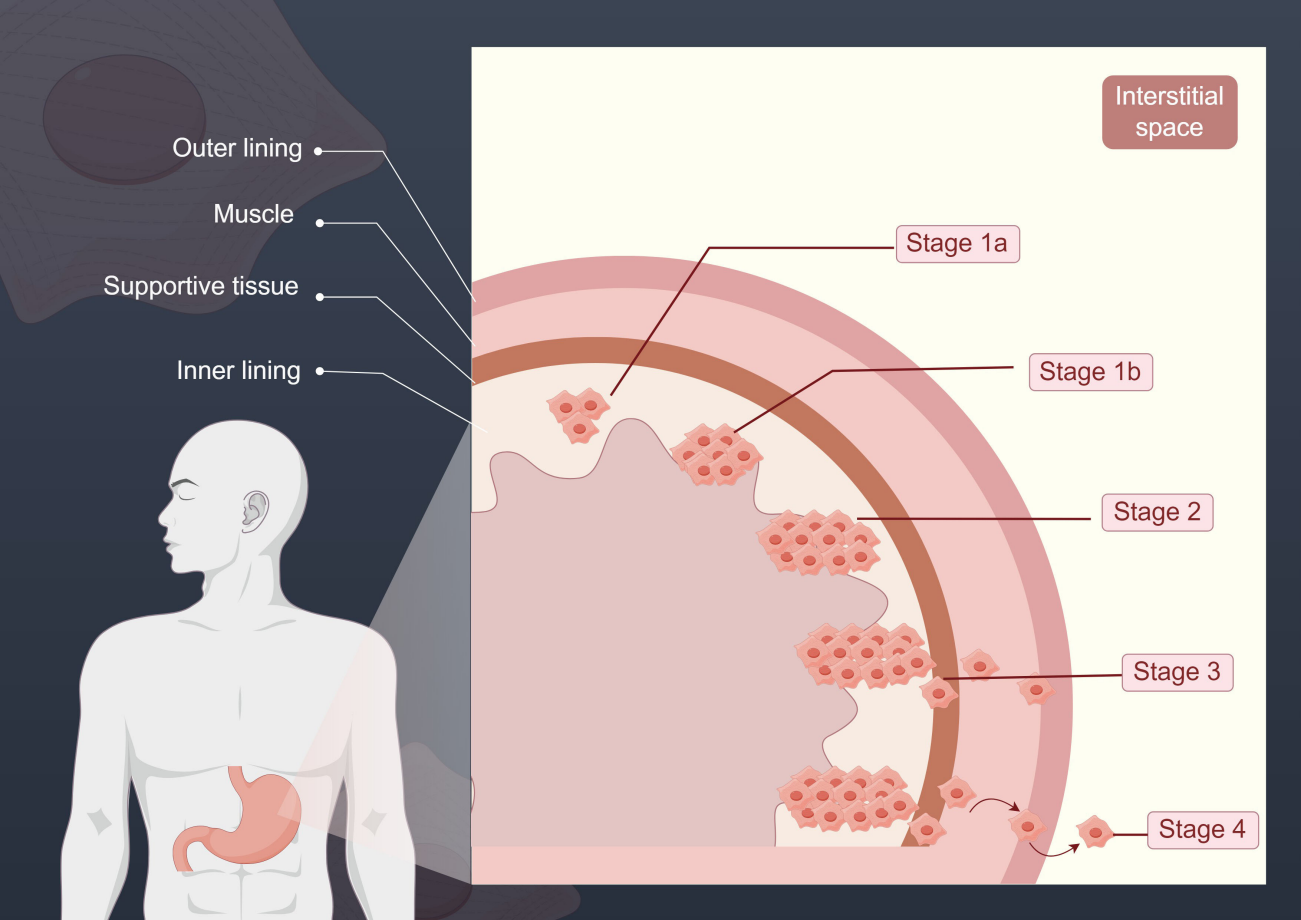
This figure illustrates the different stages of GC progression. The various stages depicted reflect the extent of cancer spread. In Stage 1a, the tumor is confined to the inner layer of the stomach; in Stage 1b, the tumor begins to breach the inner layer and invades the supporting tissue; at Stage 2, the tumor continues to expand, reaching the muscular layer; by Stage 3, the tumor has invaded more lymph nodes and extended to surrounding tissues; and in Stage 4, GC has reached its advanced stage, with the tumor metastasizing to organs distant from the primary site.

GC is one of the leading causes of cancer-related death worldwide, and its occurrence and progression involve complex molecular mechanisms. The early staging of GC is typically assessed through pathological and imaging examinations. The staging of GC is generally divided into the following stages: Stage 1a, where the tumor is confined to the inner layer of the stomach and has not yet invaded the muscular layer; Stage 1b, where the tumor has invaded the supporting tissue layer; Stage 2, where the tumor begins to invade the muscular layer and may extend to the lymph nodes; Stage 3, where the tumor invades more lymph nodes and extends to surrounding tissues; and Stage 4, the advanced stage of GC, where the tumor has metastasized to other organs such as the liver and peritoneum (58). The following figure shows the different stages of GC progression (**Figure 2**).

#### 4.1.1 Gene mutations

The development of GC is often driven by multiple gene mutations, which affect key biological processes such as cell proliferation, differentiation, apoptosis, and DNA repair. Commonly mutated genes in GC include TP53, KRAS, APC, and PIK3CA. TP53 is a tumor suppressor gene frequently mutated in GC patients. Mutations in TP53 lead to dysregulated cell cycle control, inhibition of DNA damage repair, and evasion of apoptosis, allowing cells to continue proliferating despite genetic damage, ultimately contributing to tumor formation (59). KRAS gene mutations are also an important pathogenic factor in GC. KRAS mutations activate downstream MAPK and PI3K/Akt signaling pathways, enhancing cell proliferation, migration, and invasion. These mutations play a crucial role in the initiation and progression of GC, and by altering iron metabolism mechanisms, they further promote iron accumulation, contributing to the progression of GC (6). In addition, GC is often associated with mutations in genes such as APC and PIK3CA (60, 61). APC gene mutations are typically linked to the adenocarcinoma type of GC, while PIK3CA mutations involve the activation of the PI3K/Akt pathway, further promoting the growth and metastasis of cancer cells.

In recent years, with the advancement of research, several novel candidate genes have been identified, which potentially participate in the initiation and progression of GC. Mutations in the CDH1 gene have been confirmed to be associated with genetic susceptibility to GC, particularly hereditary diffuse gastric cancer (HDGC). Such mutations in CDH1 lead to the loss or dysfunction of E-cadherin, thereby facilitating cancer cell invasion and metastasis (62). The ARID1A gene primarily participates in chromatin remodeling and transcriptional regulation, playing a crucial role in maintaining genomic stability. Recent studies have demonstrated that ARID1A mutations occur in approximately 30% of GC cases. These mutations typically result in genomic instability and are associated with therapeutic resistance, particularly in combination therapies involving immune checkpoint inhibitors (63, 64).

The initiation and progression of GC involve multiple genetic alterations that collectively disrupt key processes such as proliferation, apoptosis, metabolism, and genomic stability, driving malignancy. This complex molecular network highlights GC’s heterogeneity and underscores the need to integrate mechanisms like molecular subtyping, metabolic regulation, and epigenetic modifications to develop novel therapies and combination strategies, ultimately advancing individualized precision medicine.

#### 4.1.2 Inflammatory response

The pathogenesis of GC is intricately linked to long-term chronic inflammation, in which infection by Hp serves as a critical driving factor within this pathological cascade (65). As a class I carcinogen for GC, HP invades gastric mucosal epithelial cells directly through various virulence factors, including cytotoxin-associated gene A (CagA) protein and vacuolating cytotoxin A (VacA). These factors simultaneously activate host innate immune responses. Specifically, the CagA protein, upon translocation into host cells via a type IV secretion system (T4SS), disrupts cellular signaling pathways, resulting in the persistent activation of inflammatory cascades such as nuclear factor-kappa B (NF-κB). Consequently, this activation promotes the abundant secretion of pro-inflammatory cytokines, including interleukin-8 (IL-8) and tumor necrosis factor-alpha (TNF-α), thereby establishing and perpetuating a chronic inflammatory microenvironment (66). Meanwhile, the VacA toxin exacerbates epithelial barrier damage by disrupting mitochondrial function and inducing cellular vacuolization (67, 68). During this process, the gastric mucosa undergoes a sequential transformation from chronic non-atrophic gastritis to atrophic gastritis, intestinal metaplasia, and dysplasia, ultimately progressing to GC. In addition, HP infection can indirectly promote the formation of carcinogenic compounds such as nitrites by suppressing gastric acid secretion and altering the gastric microbiota composition, including the proliferation of nitrate-reducing bacteria (69). Notably, the synergistic interaction between host genetic susceptibility—such as cytokine gene polymorphisms—and virulent HP subtypes significantly amplifies the risk of inflammation-driven malignant transformation (70).All of these findings underscore the complexity of multifactorial interactions among the pathogen, host, and environment in gastric carcinogenesis.

#### 4.1.3 Abnormal activation of key signaling pathways

The initiation and progression of GC are closely associated with the aberrant activation of multiple key signaling pathways. Notable examples include the PI3K/Akt and MAPK/ERK pathways. The PI3K/Akt pathway plays a crucial role in the proliferation, survival, and migration of GC cells. This pathway regulates cell growth, survival, and metabolism through the activation of PI3K (phosphoinositide 3-kinase) and Akt (protein kinase B) signaling. Studies have shown that abnormal activation of the PI3K/Akt pathway not only promotes the proliferation of GC cells but also enhances their survival ability by activating downstream anti-apoptotic genes such as Bcl-2 and survivin (71). Additionally, the PI3K/Akt pathway helps cancer cells adapt to the harsh microenvironment by regulating cellular stress responses, thereby enhancing their resistance to treatment (72). Therefore, the PI3K/Akt signaling pathway has become an important target in the treatment of GC. Inhibiting this pathway may effectively suppress the growth and metastasis of GC.

The MAPK/ERK pathway is another key signaling pathway in GC, involved in processes such as cell proliferation, differentiation, stress response, and migration. This pathway is activated through members of the MAPK family (such as ERK1/2) and regulates the progression of the cell cycle, playing an important role in the invasiveness of GC cells (73). Abnormal activation of the MAPK/ERK pathway often leads to GC cells exhibiting strong migration and invasiveness at early stages (6).

The Wnt/β-catenin signaling pathway plays a pivotal role in GC, particularly in regulating cell proliferation, migration, immune evasion, and drug resistance. This pathway controls cellular proliferation, differentiation, and migration by modulating the stability of β-catenin. In GC cells, aberrant activation of Wnt/β-catenin signaling often results in uncontrolled cell proliferation and the development of therapeutic resistance. The Wnt/β-catenin pathway exerts its effects through key components such as Wnt ligands, Frizzled receptors, β-catenin, and TCF/LEF transcription factors, which collectively regulate various cellular functions (74). Activation of this pathway not only promotes cancer cell proliferation but also enhances GC cell growth and survival by upregulating downstream target genes such as MYC and Cyclin D1 (75). More importantly, aberrant activation of the Wnt/β-catenin signaling pathway is closely associated with immune evasion in cancer cells. Studies have shown that excessive activation of the Wnt/β-catenin pathway suppresses tumor immune surveillance by upregulating immune evasion–related factors, such as PD-L1 and CD47, thereby enabling tumors to escape host immune responses (76). Additionally, the Wnt/β-catenin signaling pathway is closely associated with drug resistance in GC, particularly in the context of chemotherapy and targeted therapy. Studies have revealed that activation of the Wnt/β-catenin pathway enables GC cells to resist chemotherapeutic agents by upregulating drug efflux pumps, such as MDR-1 (77).It is evident that the initiation and progression of GC are orchestrated by the multidimensional regulation of multiple key signaling pathways. These pathways form a dynamic, synergistic network that promotes GC progression by enhancing proliferation, inhibiting apoptosis, enabling immune evasion, and maintaining cancer stemness. Elucidating their crosstalk and key functional nodes may provide critical insights for targeted therapies and overcoming treatment resistance.

### 4.2 The role of ferroptosis in GC

#### 4.2.1 Iron metabolism imbalance in GC cells

Iron overload state provides unique biological characteristics that support the proliferation, metastasis, and ferroptosis of GC cells. The iron metabolism imbalance in GC cells is primarily characterized by iron accumulation and the abnormal expression of iron metabolism-related proteins (78). Key proteins involved in iron metabolism include transferrin receptor (TFR1) and ferritin, which play crucial roles in maintaining intracellular iron homeostasis. In GC cells, TFR1 is often upregulated, leading to increased iron uptake. This enhanced iron intake not only meets the needs of rapid cell proliferation but also creates favorable conditions for ferroptosis (79). Ferritin is the intracellular iron storage protein, responsible for sequestering iron ions to prevent their free form from inducing oxidative stress (80). However, GC cells attempt to store excess iron by increasing ferritin synthesis, but this storage does not effectively alleviate the accumulation of iron within the cell. On the contrary, the excessive storage of iron in ferritin may actually increase the risk of ferroptosis, as the iron stored in ferritin can still promote oxidative reactions and lipid peroxidation under certain conditions (6).

#### 4.2.2 Ferroptosis and GC cell proliferation

GC cells enhance iron uptake by upregulating the expression of TFR1 and store iron by increasing ferritin synthesis, thereby maintaining a high intracellular iron state (81). Under microenvironmental conditions such as hypoxia, GC cells activate transcription factors like hypoxia-inducible factor (HIF-1α) to further enhance iron uptake and utilization, thereby maintaining an iron overload state in low-oxygen environments. This supports the proliferation of GC cells (6). However, although iron is essential for cell proliferation, excessive iron accumulation can trigger ferroptosis, disrupt the structure and function of the cell membrane, and ultimately inhibit cell proliferation (59).

Mechanistically, iron overload serves as both a driving factor for GC cell proliferation and a potential fatal weakness. This seemingly contradictory phenomenon can be understood by recognizing the dual role of iron metabolism in tumor cells: in the early stages of GC cell proliferation, iron overload promotes rapid cell growth. However, as the degree of iron overload deepens, excessive ROS production and increased oxidative stress ultimately lead to ferroptosis, inhibiting further proliferation. Thus, iron overload is both a necessary condition for GC proliferation and a triggering factor for ferroptosis. By using ferroptosis inducers, it is possible to enhance iron accumulation and ROS levels in GC cells, thereby triggering ferroptosis and suppressing cell proliferation (71).

In recent years, increasing research has focused on the upstream regulatory axes of ferroptosis in GC, particularly the identification of the USP7/SCD axis, which provides a molecular basis for understanding the link between ferroptosis and cell proliferation. Ubiquitin-specific protease 7 (USP7) is a key deubiquitinating enzyme and has been shown to stabilize stearoyl-CoA desaturase 1 (SCD1) protein levels through its deubiquitinating activity. SCD1 catalyzes the synthesis of monounsaturated fatty acids (MUFAs), thereby reducing the proportion of oxidizable polyunsaturated fatty acids (PUFAs) in membrane lipids and decreasing cellular sensitivity to ferroptosis (82, 83). *In vitro*, the USP7 inhibitor DHPO significantly reduced the proliferative activity of GC cells and induced lipid peroxidation and iron accumulation, exhibiting hallmark features of ferroptosis. *In vivo*, in a xenograft mouse model, DHPO treatment significantly reduced tumor volume without observable signs of systemic toxicity, suggesting that USP7 functions as a ferroptosis regulator and may represent a promising therapeutic target (82). Moreover, SCD1 is also highly expressed in GC stem cells and enhances cholesterol biosynthesis by relieving p53-mediated transcriptional repression of squalene epoxidase (SQLE), thereby activating the mTOR signaling pathway. This mechanism not only maintains the stemness of GC stem cells, but also enhances their resistance to ferroptosis. Animal studies have shown that SCD1 knockdown or inhibition of cholesterol uptake significantly increases the sensitivity of GC stem cells to ferroptosis inducers, further suppressing their proliferative capacity (83). These findings highlight the USP7/SCD1 axis as a critical regulatory mechanism of ferroptosis and suggest that targeting this axis may enhance the anti-proliferative effect of ferroptosis on GC cells. This provides a potential molecular target for therapeutic intervention.

Mechanistically, iron overload acts both as a driver of GC cell proliferation and as a potential vulnerability. On one hand, to sustain rapid proliferation, cancer cells actively reprogram iron metabolism pathways by upregulating iron import proteins such as TFR1 and DMT1, while downregulating the FPN, thereby increasing intracellular Fe²^+^ uptake, which in turn promotes mitochondrial function, DNA synthesis, and cell cycle progression (84). At this stage, moderate iron accumulation does not pose a threat but rather serves as a key driver for maintaining cellular viability and enhancing metabolic activity in cancer cells. However, excessive iron induces ferroptosis (85). This process has been experimentally validated to exert significant antitumor effects in GC. Studies have shown that the natural compound Polyphyllin I induces Fe²^+^ accumulation and lipid ROS production, inhibits the NRF2-mediated antioxidant pathway, thereby effectively activating ferroptosis in GC cells, resulting in pronounced tumor-suppressive effects (86).

**Figure 3:**
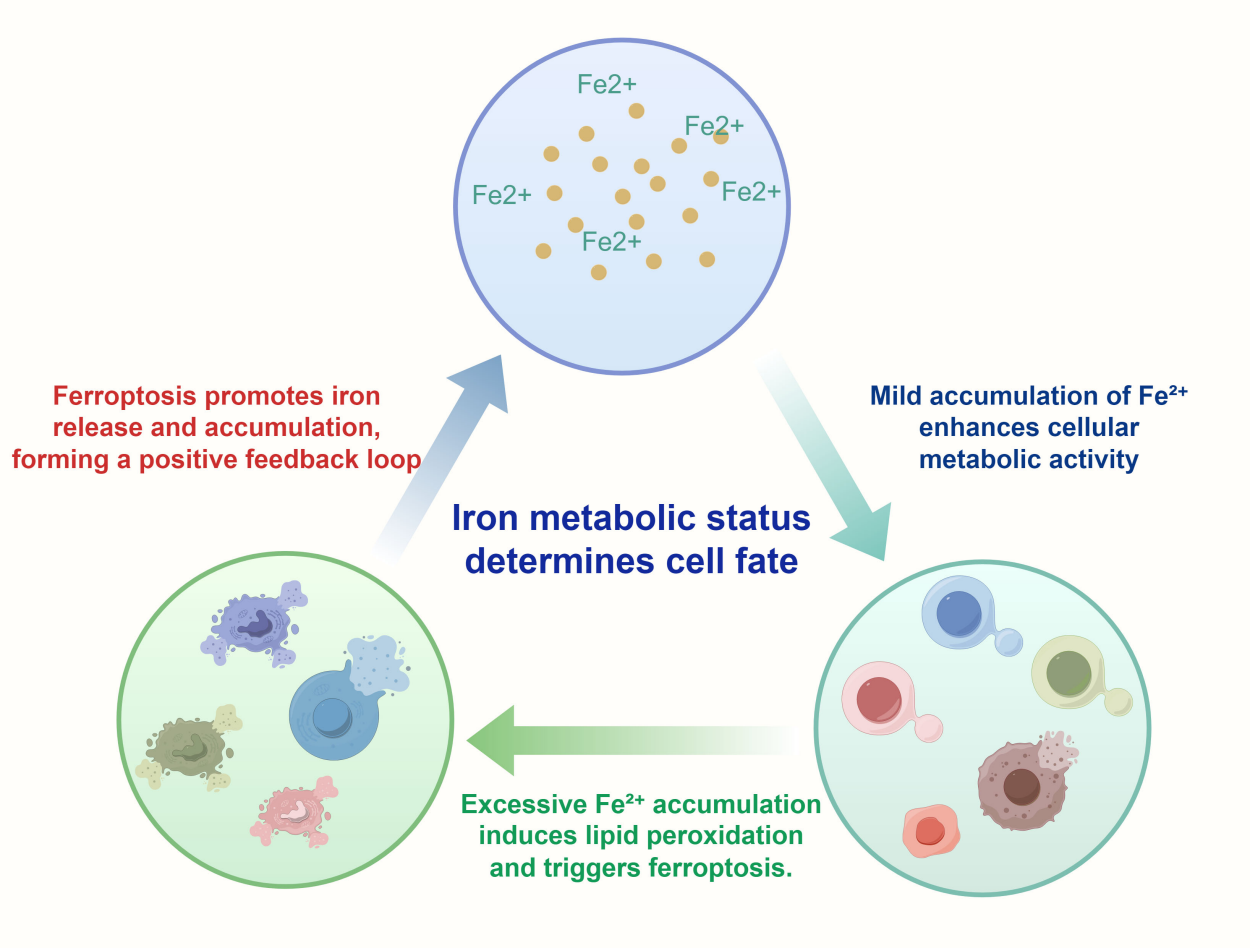
This Figure illustrates how iron metabolism dynamically influences cell fate decisions. It depicts the distinct effects of Fe²^+^ at varying intracellular concentrations on cellular functions: Mild Fe²^+^ accumulation enhances metabolic activity, thereby promoting cell proliferation and survival (87). In contrast, excessive accumulation of Fe²^+^ induces lipid peroxidation and mitochondrial damage, ultimately triggering ferroptosis (88). At high intracellular concentrations, Fe²^+^ participates in the Fenton reaction, generating hydroxyl radicals (•OH) that initiate lipid peroxidation cascades. This lipid peroxidation primarily affects PUFAs within membrane phospholipids, leading to the accumulation of lipid hydroperoxides (PUFA-OOH), which are cytotoxic when not neutralized by antioxidant systems such as GPX4 (89). Importantly, ferroptosis itself can further release Fe²^+^, forming a positive feedback loop that exacerbates intracellular iron burden and oxidative stress (90). The entire illustration provides a visual representation of the delicate balance of iron metabolism between cell survival and death, particularly in tumor cells.

Therefore, dysregulated iron metabolism not only drives the continuous proliferation of GC cells but also provides a potential therapeutic target. By modulating iron metabolic pathways or applying ferroptosis inducers to activate this programmed cell death process, it is possible to achieve precise and effective treatment of GC. The following figure illustrates how the state of iron metabolism influences distinct cellular fates (**Figure 3**).

#### 4.2.3 Ferroptosis and GC cell metastasis

Metastasis is a critical determinant of prognosis and therapeutic strategy in GC. It involves the dissemination of cancer cells from the primary tumor to distant tissues and organs, ultimately forming metastatic lesions. This highly invasive and complex process commonly occurs via hematogenous and lymphatic routes, with frequent metastases to the liver and peritoneum, substantially complicating treatment and reducing patient survival. Emerging evidence indicates that GC cells often exhibit iron overload, which can induce ferroptosis, compromise cell membrane integrity, and consequently inhibit the migratory and invasive capacities of GC cells. Studies have shown that ferroptosis effectively limits the metastatic potential of GC cells through this mechanism (59, 91). At the same time, the excessive production of ROS also affects the cytoskeletal structure of GC cells. By disrupting the stability of microtubules and microfilaments, it alters cell morphology and motility. These changes cause GC cells to lose their ability to migrate during the metastasis process, preventing them from effectively invading distant tissues (92). However, iron overload during ferroptosis also activates multiple signaling pathways associated with metastasis, such as the MAPK/ERK and PI3K/Akt pathways, which enhance the proliferation, migration, and invasiveness of GC cells (93, 94). Meanwhile, ferroptosis also regulates cell migration by modulating GSH metabolism. ATP-binding cassette subfamily C member 2 (ABCC2) can be induced by oxidative stress in various cancer cells, enhancing its capacity for GSH efflux, thereby promoting lipid ROS accumulation and increasing cellular sensitivity to oxidative stress and ferroptosis (95). Studies have found that ABCC2 is highly expressed in GC cell lines with low metastatic potential, suggesting that it may play an inhibitory role in modulating ferroptosis sensitivity and limiting cell migration (96).

**Figure 4:**
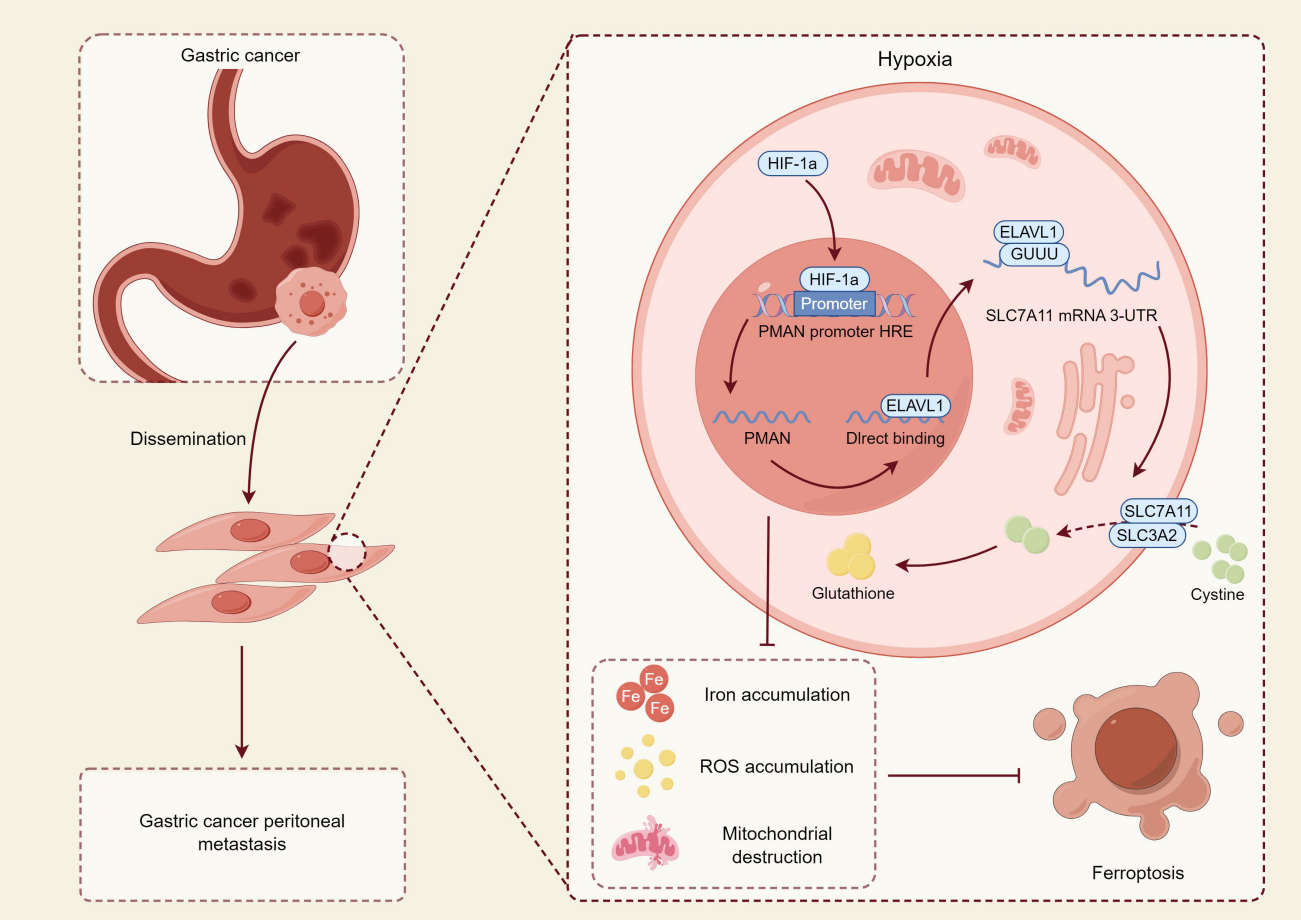
This figure illustrates the molecular mechanism by which GC cells regulate their survival and metastatic potential under hypoxic conditions through modulation of the ferroptosis pathway. In the hypoxic tumor microenvironment, hypoxia-inducible factor 1α (HIF-1α) is upregulated, activating the hypoxia response element (HRE) region of the promoter of PMAN, a hypoxia-associated long non-coding RNA, thereby promoting its transcription (97). Upregulated PMAN directly binds to and stabilizes ELAVL1 (HuR), an RNA-binding protein that functions as a post-transcriptional stabilizer, which recognizes and binds to the 3′-untranslated region (3′-UTR) of SLC7A11 mRNA, enhancing its stability and translational efficiency (98). SLC7A11 and SLC3A2 together form system Xc^-^, which facilitates cystine uptake in tumor cells for the synthesis of GSH, thereby eliminating ROS and suppressing ferroptosis (14, 99). Activation of this pathway confers enhanced antioxidant capacity to tumor cells, enabling their survival in hostile microenvironments and promoting metastatic potential. In contrast, in certain cells where this antioxidant axis is insufficiently activated, Fe²^+^ and ROS accumulate, leading to mitochondrial destruction and elevated lipid peroxidation, ultimately inducing ferroptosis and exerting antitumor effects (100). This mechanism suggests that ferroptosis plays a dual role in GC—both tumor-suppressive and pro-metastatic: while functioning as a cell death pathway to limit tumor expansion, its adaptive escape mechanisms may conversely enhance metastatic potential.

However, despite these insights, the current understanding of ferroptosis in GC metastasis remains fragmented and, in some cases, contradictory. While *in vitro* studies have demonstrated both inhibitory and promotive effects on migration and invasion, comprehensive *in vivo* validations are still lacking. Moreover, the context-dependent nature of ferroptosis—whereby factors such as ROS levels, iron homeostasis, and the tumor microenvironment intricately modulate its dual role—has yet to be fully elucidated. Therefore, further mechanistic investigations and well-designed preclinical studies are essential to clarify how ferroptosis can be precisely manipulated to suppress metastasis without triggering unintended pro-metastatic signals. Such efforts will be pivotal in translating ferroptosis-targeting strategies into clinically viable interventions for metastatic GC. The following figure illustrates how the ferroptosis mechanism influences the survival and metastasis of GC cells (**Figure 4**).

### 4.3 Common programmed cell death pathways in GC

Cell death is a fundamental biological process essential for maintaining tissue homeostasis and suppressing tumor initiation and progression. In GC, multiple forms of programmed cell death act cooperatively or competitively to regulate tumor cell fate, directly influencing therapeutic response and prognosis. Among them, apoptosis, necroptosis, pyroptosis, and ferroptosis represent the four most intensively studied cell death pathways to date.

#### 4.3.1 Apoptosis

Apoptosis is a classical form of programmed cell death, characterized by preserved membrane integrity, cell shrinkage, chromatin condensation, and DNA fragmentation (101). Caspases (cysteine-aspartic proteases) are a family of cysteine-dependent proteases that specifically cleave aspartic acid residues, and are broadly involved in programmed cell death. Different types of cell death exhibit varying degrees of dependence on caspases, thereby making them a key criterion for distinguishing between different cell death pathways (102). Apoptosis is primarily mediated through the caspase-3, caspase-9, and Bcl-2/Bax signaling pathways. Many chemotherapeutic agents, such as cisplatin, paclitaxel, and 5-fluorouracil (5-FU), exert antitumor effects by inducing apoptosis (103, 104). However, GC cells frequently exhibit p53 mutations or Bcl-2 overexpression, resulting in reduced sensitivity to apoptotic stimuli, which contributes to the development of therapeutic resistance (105).

#### 4.3.2 Necroptosis

Necroptosis is a form of regulated necrotic cell death, characterized by plasma membrane rupture and the release of proinflammatory cytokines, and is typically mediated by signaling pathways involving receptor-interacting protein kinase 1 (RIPK1), RIPK3, and mixed lineage kinase domain-like protein (MLKL). It can induce effective cell death and stimulate immune responses in apoptosis-resistant GC cells, particularly by promoting the activation of dendritic cells (DCs) and cytotoxic cluster of differentiation 8-positive T cells (CD8^+^ T cells) through the release of damage-associated molecular patterns (DAMPs). However, excessive activation of necroptosis may lead to immunosuppression or enhanced metastasis through the upregulation of inflammatory mediators such as tumor necrosis factor-α (TNF-α) and interleukin-8 (IL-8) (106, 107).

#### 4.3.3 Pyroptosis

Pyroptosis is a form of programmed cell death that is dependent on inflammasome activation—such as nucleotide-binding oligomerization domain-like receptor family pyrin domain-containing 3 (NLRP3)—and caspase-1. It is characterized by the cleavage of gasdermin D (GSDMD), which forms pores in the plasma membrane, leading to cell swelling and the release of cytoplasmic contents. While pyroptosis is well established in antibacterial immunity, recent studies have extended its relevance to GC, suggesting that it may contribute to differential chemotherapy responses and immune activation. In particular, the upregulation of interleukin-1β (IL-1β) and interleukin-18 (IL-18) has been closely associated with patient prognosis (108).

#### 4.3.4 Ferroptosis

Ferroptosis, the primary focus of this review, is a recently identified form of cell death that is mechanistically distinct from the aforementioned pathways. It is characterized by iron accumulation, lipid peroxidation, and dysfunction of the antioxidant defense system. GC cells often exhibit dysregulated iron metabolism, making them susceptible to ferroptotic regulation. Ferroptosis is unique in that it is caspase-independent, capable of inducing immunogenic cell death, and able to overcome apoptosis resistance, making it a promising next-generation therapeutic target (109). Among the aforementioned cell death modalities, ferroptosis is morphologically most similar to necroptosis, but it can be clearly distinguished based on characteristic biomarkers such as 4-HNE, MDA, and changes in GPX4 expression. Studies have also shown that ferroptosis does not rely on cytochrome c release or DNA fragmentation, indicating that conventional apoptotic markers are not applicable to its detection (110). The table below provides a comparative overview of the four major forms of cell death, summarizing their key induction mechanisms, morphological features, and associated signaling pathways. The table below provides a comparative summary of the key induction mechanisms, morphological characteristics, and signaling axes of the four major forms of cell death (**Table 2**).

**Table 2:** Comparative analysis of mechanistic differences and key signaling pathways among distinct forms of programmed cell death.

Cell Death Type	Induction Mechanism	Morphological Features	Caspase Dependence	Key Molecules and Signaling Axis	References
Ferroptosis	Fe²^+^ overload, lipid ROS, GPX4/FSP1 inactivation	Condensed mitochondria, dense membranes, no nuclear fragmentation	No	GPX4、ACSL4、SLC7A11、FSP1	(105)
Apoptosis	Cytochrome c release; Bcl-2 axis	Chromatin/DNA condensation; membrane blebbing	Yes	Caspase-3/8/9、Bcl-2、p53	(103)
Necroptosis	RIPK1/RIPK3 activation; MLKL membrane insertion	Cell swelling, membrane rupture, and release of cytoplasmic contents	No	RIPK1、RIPK3、MLKL	(111)
Pyroptosis	Inflammasome activation and caspase-1–mediated GSDMD cleavage	Cell swelling, membrane pore formation by GSDMD, and release of inflammatory cytokines	Yes	NLRP3、Caspase-1、GSDMD、IL-1β/IL-18	(108)

## 5 Applications of ferroptosis in GC

### 5.1 Inhibition of cancer cell proliferation and metastasis

A research group used single-cell RNA and TCR/BCR sequencing on samples from 20 treatment-naive metastatic GC patients, revealing notable overexpression of Gpx4, a key ferroptosis regulator, in cancer cells. High Gpx4 levels hindered ferroptosis, correlated positively with macrophage abundance, and negatively with patient prognosis. Notably, Gpx4 was the sole overexpressed ferroptosis regulator in GC cells. Further experiments showed that Gpx4 knockout suppressed tumor growth, slowed proliferation, increased cell death, and prevented distant metastases *in vivo*. Inhibiting Gpx4 or its upstream pathways produced similar effects and synergized with CAR-T therapy to enhance antitumor efficacy (112). A study identified a natural small molecule, DHPO, as an inhibitor of USP7, which can induce ferroptosis in GC cells by inhibiting USP7-mediated targeted degradation of stearoyl-CoA desaturase (SCD). In *in vitro* experiments, DHPO significantly suppressed GC cell growth and metastasis. In an orthotopic tumor mouse model, DHPO also demonstrated potent anti-tumor effects without significant toxicity (82).

In recent years, as understanding of ferroptosis has advanced, ferroptosis inducers like Erastin and RSL3 have gained attention for their translational potential in GC. While early research focused on cell lines and murine models, recent studies are addressing challenges such as poor pharmacokinetics and limited specificity. A 2024 study summarized the clinical prospects of ferroptosis inducers across solid tumors, noting that structurally optimized compounds showed improved metabolic stability, enhanced antitumor efficacy *in vivo*, and better tumor targeting in GC through nanodelivery systems, marking steady progress toward clinical application (113). Erastin and RSL3 have demonstrated potent antitumor activity in multiple human tumor xenograft models, including various solid tumors such as GC. In a study using a cisplatin-resistant GC xenograft model, the combination of Erastin and cisplatin significantly enhanced tumor suppression and improved immune cell infiltration profiles, suggesting its potential to modulate the immunological microenvironment in GC (114).

These advances have undoubtedly accelerated the exploration of ferroptosis in GC therapy, yet they also underscore the paradoxical challenges inherent to this field. While targeting Gpx4 and its associated signaling axes offers promising therapeutic avenues, ferroptosis exhibits high plasticity across different cellular subpopulations and microenvironmental contexts, making its controllability a critical bottleneck for clinical translation. It remains unclear whether ferroptosis induction within complex immune niches might trigger compensatory escape mechanisms, or even lead to “ferroptosis tolerance” in certain tumor clones. Moreover, the delivery and bioactivity of ferroptosis inducers continue to be constrained by the precision and biocompatibility of nanocarrier systems. Thus, future efforts must move beyond the question of how to induce ferroptosis efficiently, and focus on the deeper challenge of when, where, and in whom to induce it—an essential step toward transforming ferroptosis from a laboratory phenomenon into a controllable clinical tool.

### 5.2 Overcome GC drug resistance

GC, a leading cause of cancer mortality worldwide, is plagued by chemotherapy resistance, which significantly limits treatment success. Despite advances in drug development, resistant tumors remain a major obstacle. Ferroptosis, an iron-dependent form of programmed cell death driven by oxidative stress and lipid peroxidation, has shown promising potential to overcome drug resistance in GC.

Studies have demonstrated that ferroptosis inducers such as Erastin and RSL3 significantly enhance the sensitivity of GC cells to cisplatin, offering a promising approach for overcoming chemoresistance (115). Meanwhile, studies have shown that the combination of FIN56 and cisplatin can overcome drug resistance in GC cells, significantly enhancing their sensitivity to cisplatin and providing a new strategy for the treatment of drug-resistant gastric cancer (8, 116). Further studies have confirmed that the combination of RSL3 and cisplatin significantly inhibits GC cell growth and overcomes drug resistance. This strategy has also demonstrated favorable outcomes in other tumor types, indicating broad therapeutic potential (116–118).

Many drug-resistant cancers, including GC, develop resistance in part due to the activation of immune escape mechanisms, which significantly reduce the effectiveness of chemotherapy and immunotherapy. Cancer cells exploit these immune escape mechanisms to evade immune system attacks, thereby enhancing their resistance to chemotherapy drugs (119, 120). In the TME, immunosuppressive factors such as PD-L1, TGF-β, and IL-10 suppress immune cell activity and promote immune evasion. Ferroptosis inducers increase ROS production, kill tumor cells, and reduce these factors’ expression, thereby remodeling the TME and enhancing anti-tumor immunity (121, 122). Ferroptosis inducers such as Erastin and FIN56 can enhance the antitumor immune response against GC, indicating potential immunomodulatory effects (123, 124). Studies have shown that when Erastin is combined with immune checkpoint inhibitors (such as anti-PD-1 antibodies), it can significantly enhance the anti-immune escape effect of tumors, helping the immune system better recognize and eliminate cancer cells (125).These mechanisms provide new directions for addressing the issue of drug resistance in GC.

These findings suggest that ferroptosis inducers not only exert direct cytotoxic effects on tumor cells but also reshape the tumor immune microenvironment, establishing a novel therapeutic paradigm of combined cytotoxic and immunomodulatory action. This indicates that the role of ferroptosis in drug-resistant malignancies like GC is evolving from a single cell death pathway to an integrated “chemo–immune” strategy. It underscores the need for future research to focus on the precise modulation of ferroptosis within complex tumor microenvironments and to explore deeper synergy with immunotherapy. Such an approach holds promise for overcoming the limitations of conventional therapies and advancing toward personalized, multidimensional anticancer strategies.

### 5.3 As a biomarker for GC

To facilitate the application of ferroptosis mechanisms in precision therapy, recent studies have focused on the expression profiles and clinical relevance of associated molecular biomarkers in patient populations. Ferroptosis biomarkers include products associated with lipid peroxidation, iron metabolism, and ROS generation. These biomarkers not only reflect the iron metabolic status in GC but also provide a strong basis for early cancer screening. As a key antioxidant enzyme responsible for maintaining intracellular lipid homeostasis, GPX4 has been validated by multiple studies for its expression patterns and prognostic value in GC. Studies have shown that GPX4 expression is significantly elevated in GC tissues compared to matched normal tissues, and its expression levels are negatively correlated with lymph node metastasis, tumor aggressiveness, and overall prognosis, suggesting that GPX4 not only participates in the regulation of GC biological behavior but may also serve as a potential prognostic biomarker (126, 127).

In addition to GPX4, lipid peroxidation products such as 4-HNE and MDA, which are key metabolites in ferroptosis, have also emerged as focal points of research. In a prospective cohort study, researchers detected a significant correlation between 4-HNE levels and TNM staging in plasma and tissue samples from GC patients. Moreover, elevated levels of 4-HNE were observed in patients with postoperative recurrence, suggesting its potential as a dynamic biomarker for monitoring GC progression and recurrence. The study also confirmed a positive correlation between 4-HNE levels and the infiltration of M2 macrophages in the tumor immune microenvironment, further elucidating its involvement in mechanisms of tumor immune evasion (128).

Research has shown that USP7 (ubiquitin-specific protease 7), as a driver gene in GC, plays a key role in the regulation of ferroptosis. By modulating the stability of stearoyl-CoA desaturase (SCD), USP7 inhibits ferroptosis, thereby promoting GC growth and metastasis. Therefore, USP7 not only serves as a potential prognostic biomarker for GC but also provides a new avenue for targeted therapy based on ferroptosis regulation (82). Another research team found that high expression of ABCC2 is associated with a better prognosis in GC patients. ABCC2 affects intracellular amino acid metabolism and redox status by exporting GSH, thereby increasing GC cell susceptibility to ferroptosis. Further experimental data showed that GC cells with high ABCC2 expression exhibited significant biological changes under conditions of amino acid deprivation and ferroptosis induction. This suggests that ABCC2 not only serves as a prognostic biomarker for GC but also as a potential target for targeted ferroptosis therapy (129). This study highlights the unique value of ABCC2 as a ferroptosis biomarker in GC, providing important evidence for its potential as a tool for predicting GC prognosis and treatment response.

### 5.4 Clinical progress and translational challenges of ferroptosis-targeted therapy

In recent years, multiple registered clinical trials focusing on ferroptosis mechanisms have been initiated worldwide. Although no dedicated studies targeting gastric cancer have been reported to date, preliminary explorations have been conducted in various solid tumors, including hepatocellular carcinoma, breast cancer, and lung cancer. Derivatives of ferroptosis inducers such as Erastin and RSL3 are currently being evaluated in several phase I clinical trials for their safety and pharmacokinetic profiles, with some studies investigating their synergistic effects in combination with chemotherapy or immune checkpoint inhibitors (130). Additionally, FSP1 inhibitors, as novel non-GPX4-dependent targeted agents, have demonstrated potential to overcome drug resistance in prospective studies of breast cancer and non-small cell lung cancer (131). Moreover, certain nanocarrier delivery systems have also been introduced into early-phase clinical studies, aiming to enhance therapeutic efficacy through precise delivery (132).

Currently, preclinical studies on ferroptosis mechanisms are steadily advancing, with numerous *in vitro* and animal model experiments laying the groundwork for clinical translation. In clear cell renal cell carcinoma studies, the mTOR inhibitor Everolimus has been shown to significantly enhance ferroptosis induced by Erastin and RSL3, through a synergistic mechanism that promotes lipid peroxidation and ROS accumulation, which holds promise for overcoming resistance to mTOR monotherapy (133). Moreover, in triple-negative breast cancer (TNBC), studies have shown that resistance to RSL3-induced ferroptosis can be significantly reversed by the irreversible HER2 inhibitor Neratinib, and this effect is dependent on Neratinib-induced mitochondrial ROS production and DNA replication stress, thereby increasing cellular sensitivity to ferroptosis (134). This study provides a rationale for combination therapy strategies in future clinical trial design. In prostate cancer models, the combination of ferroptosis inducers with the conventional chemotherapeutic agent paclitaxel has also shown promising results. RSL3 and Erastin significantly enhance paclitaxel-induced apoptosis by downregulating GPX4 expression, suggesting their potential application in overcoming chemotherapy resistance (135). This finding provides a theoretical basis for the synergistic development of ferroptosis mechanisms and standard chemotherapy. Regarding non-GPX4-dependent pathways, a study on lung adenocarcinoma revealed that knockdown of nicotinamide adenine dinucleotide kinase (NADK) can suppress FSP1 expression by reducing NADPH levels, thereby enhancing the cytotoxicity of Erastin and RSL3, highlighting the critical role of the NADK-FSP1 axis in regulating ferroptosis (136). In ovarian cancer models, the highly expressed adhesion molecule JAM3 can induce ferroptosis resistance by activating the NRF2/FSP1 signaling axis, providing important rationale for the use of FSP1 inhibitors as reversal agents (136). While ferroptosis-targeted clinical research in gastric cancer remains absent, systematic investigations of Erastin, RSL3, and the FSP1 pathway have yielded promising results in various solid tumors, establishing a preclinical research framework that integrates mechanisms, pharmacological agents, and combination strategies. These achievements provide a solid theoretical foundation and a feasible roadmap for future clinical translation of ferroptosis-targeted therapies.

Despite these promising advances, the clinical translation of ferroptosis-targeted therapies still faces significant obstacles. Firstly, although various candidate agents have shown excellent performance in cellular and animal models, clear clinical benefit data are still lacking, and, in particular, no ferroptosis inducers have yet been approved to advance into phase II/III clinical trials. Secondly, the *in vivo* distribution and pharmacokinetics of these agents are complex, and clinical trials often encounter issues such as uneven drug retention and dose-limiting toxicities, which remain critical barriers to practical application (137, 138). Moreover, there is currently a lack of well-established biomarkers for ferroptosis, making it difficult to monitor in real time whether therapeutic agents have successfully activated ferroptosis pathways in clinical settings, which constitutes one of the major bottlenecks in clinical trial design (139). Although ferroptosis-targeted therapy has shown cross-cancer potential, the fundamental bottleneck lies in the incomplete “validation chain” from mechanistic insight to clinical efficacy. The current lack of real-time dynamic biomarkers and precise delivery systems prevents the formation of a true translational loop. Future breakthroughs will depend not on single-agent advances but on the development of integrated systems that combine mechanism, monitoring, and intervention—this will be the decisive path for ferroptosis to achieve meaningful clinical application.

## 6 The dual role of ferroptosis in GC treatment

### 6.1 Potential side effects of ferroptosis-targeted therapy in GC

In recent years, ferroptosis has been regarded as highly promising in the treatment of GC. On one hand, it can induce programmed death of cancer cells; on the other hand, due to its extensive overlap with fundamental biological processes, insufficient selectivity may cause potential damage to normal tissues and even the immune system, exhibiting a typical “double-edged sword” effect.

Ferroptosis inducers can inhibit xCT or GPX4, thereby reducing cellular antioxidant capacity and triggering a burst of ROS and iron ions. However, such mechanisms are not exclusive to cancer cells; highly metabolic tissues such as hepatocytes, renal tubular epithelial cells, and cardiomyocytes are also sensitive to lipid peroxidation. Therefore, ferroptosis-inducing therapies carry the risk of non-specific organ toxicity (140). In a murine model, feeding with a high-iron diet or genetic predisposition to systemic iron overload resulted in more than a twofold increase in serum ALT levels and a significant elevation of hepatic MDA content. Treatment with Ferrostatin-1 markedly reduced ALT levels and restored MDA concentrations to near-control levels, indicating that iron overload-induced liver injury is indeed mediated by ferroptosis and can be alleviated by ferroptosis inhibitors (141).A 2024 study using a murine liver model demonstrated that Erastin-induced ferroptosis not only suppressed tumor progression but also led to significant hepatotoxic effects, including elevated ALT levels and accumulation of lipid peroxidation products, Administration of Ferrostatin-1 effectively alleviated these toxicities, suggesting that ferroptosis induction may exert dose-dependent systemic side effects (142). Moreover, cardiomyocytes are highly sensitive to ferroptosis due to their abundant mitochondria, active metabolism, and high content of unsaturated fatty acids. Studies have indicated that prolonged or high-dose administration of ferroptosis inducers may result in arrhythmias and structural damage to cardiac tissue, thereby limiting their application in systemic treatment of solid tumors (143, 144).

In addition to organ toxicity, ferroptosis induction may indirectly promote tumor metastasis and immune suppression through inflammatory cascade reactions. A study demonstrated that, in a hepatocellular carcinoma model, ferroptotic cells released oxidized phospholipids and DAMPs (such as HMGB1 and 4-HNE), which activated the NLRP3 inflammasome in macrophages. This process promoted IL-1β secretion, triggered neutrophil recruitment and vascular remodeling, and ultimately accelerated the formation of pulmonary metastases (145). This suggests that while ferroptosis exerts cytotoxic effects within the tumor core, insufficient or heterogeneous induction at the tumor margins may paradoxically promote immune evasion and the development of micrometastases through inflammation-mediated mechanisms. Although ferroptosis is considered immunogenic and capable of inducing the release of DAMPs to activate antitumor immune responses, recent studies have shown that ferroptosis inducers may suppress the function of CD8^+^ T cells and NK cells under certain conditions, thereby reducing the efficacy of immunotherapy. This effect is particularly pronounced within the tumor microenvironment, where improper dosing may create an “immunosuppressive window” that antagonizes the efficacy of immune checkpoint inhibitors (146).

**Figure 5:**
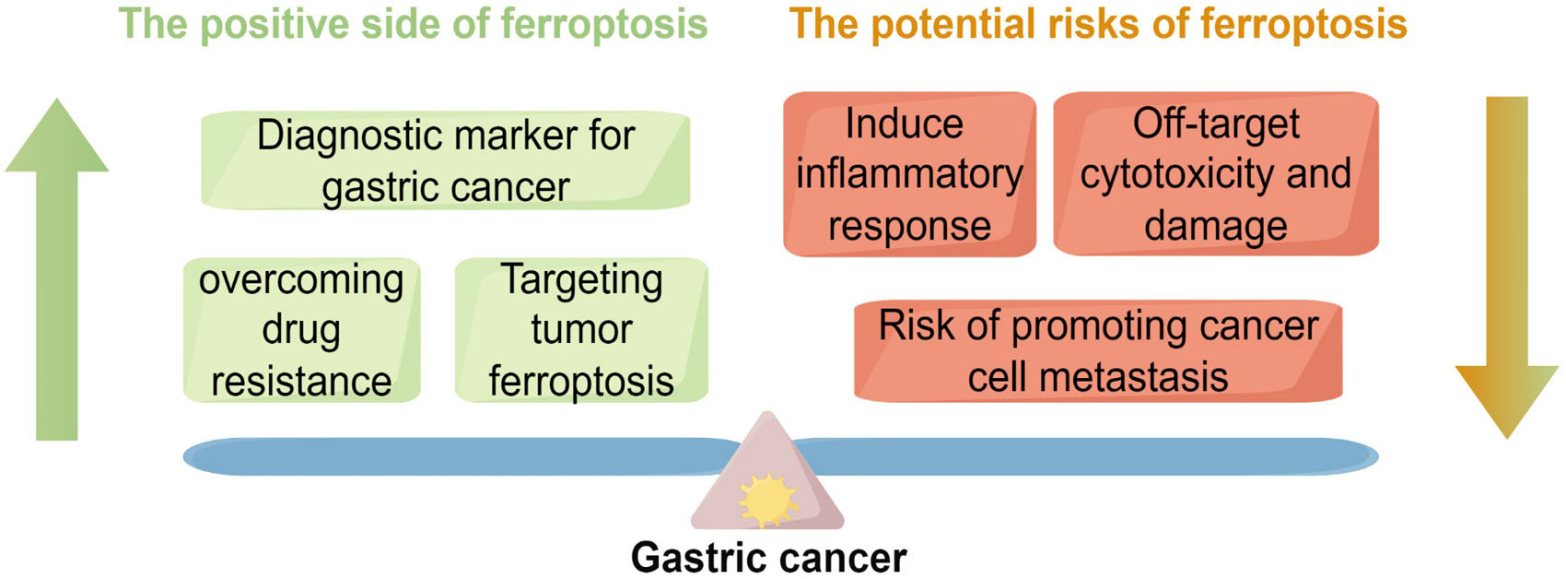
This figure illustrates the dual role of ferroptosis in GC,underscoring the complexity of its regulatory mechanisms. On theone hand, ferroptosis, as an emerging form of cell death, exhibitsnotable therapeutic potential in GC. For example, ferroptosis canserve as a diagnostic biomarker for early cancer detection; it may alsoovercome drug resistance and provide new therapeutic strategies bytargeting tumor ferroptosis mechanisms. These positive effects opennew avenues for GC treatment. On the other hand, the figure alsopoints out the potential risks of ferroptosis, such as triggeringunnecessary inflammatory responses, causing off-target cytotoxicdamage, and possibly promoting cancer cell metastasis.

In summary, ferroptosis has demonstrated substantial potential in overcoming GC drug resistance and enhancing antitumor immunity. However, its associated side effects are not solely attributable to drug toxicity but also reflect intrinsic limitations of the ferroptosis mechanism itself—it can effectively kill cancer cells but may likewise damage normal high-metabolic tissues and immune cells. This underscores that selectivity remains the central challenge currently facing ferroptosis-based therapies. Only by successfully achieving a balance between cytotoxic efficacy and safety can ferroptosis be translated from experimental research into a safe and effective clinical application. The following figure illustrates the dual potential of ferroptosis in GC treatment (**Figure 5**).

### 6.2 Mitigation strategies

#### 6.2.1 Nanodelivery platforms

Nanomaterials possess favorable biodegradability, high drug-loading capacity, and versatile surface modifiability, making them ideal carriers for the delivery of ferroptosis inducers. By modifying the surface of nanoparticles, researchers can enable them to respond to specific physiological features of the GC microenvironment, such as acidic pH, elevated ROS levels, and high esterase activity, thereby achieving an “activation-and-release” profile in tumor tissues while remaining “dormant” in normal tissues. A research team developed Fe_3_O^4^@PDA@Erastin magnetic-responsive nanoparticles that release Erastin by triggering the degradation of the PDA shell in response to elevated H_2_O_2_ levels within the tumor region, thereby inducing ferroptosis in tumor cells. In a murine GC model, this system exhibited potent antitumor efficacy with no significant hepatotoxicity or nephrotoxicity (113). The rise of nanodelivery systems fundamentally reflects the high precision required across the entire chain of ferroptosis therapy—encompassing delivery, activation, and regulation. Unlike conventional drugs, ferroptosis induction not only necessitates transporting agents to the tumor site but, more critically, demands precise activation of cytotoxic effects under specific microenvironmental conditions; otherwise, off-target damage is likely to occur. This highlights a key insight: the future of ferroptosis therapy must shift from mere physical delivery to biologically driven, dynamic sensing systems.

#### 6.2.2 Intermittent induction and dose optimization

Although ferroptosis holds the potential to induce immunogenic cell death (ICD), its intense lipid peroxidation and uncontrolled release of intracellular components, if not temporally regulated, may lead to excessive inflammation, immune cell exhaustion, or organ damage (147). Therefore, precise spatiotemporal regulation of ferroptosis induction is crucial to achieving clinical controllability and therapeutic efficacy. The “low-dose–high-frequency” model enables mild modulation of ferroptotic activity. Compared with high-dose, instantaneous induction, this strategy enables sustained release of DAMPs and oxidized lipid signals, which promotes dendritic cell (DC) activation and antigen presentation, thereby enhancing CD8^+^ T cell activation and tumor recognition (148). Studies have shown that intermittent administration of Erastin in combination with anti-PD-1 therapy can enhance immune responses without significantly inducing hepatotoxicity or nephrotoxicity. It effectively increases T cell activity, IFN-γ secretion, and tumor suppression, demonstrating superior synergistic immune activation compared to monotherapy or continuous high-dose regimens (149). Moreover, intermittent induction may also contribute to the establishment of immunological memory. A 2024 study demonstrated that oral administration of low-dose ferroptosis inducers combined with anti-PD-1 therapy not only completely suppressed the primary colorectal tumor, but also conferred 100% immune protection in rechallenge models, highlighting its strong potential in inducing long-term memory responses (150). This suggests that maximizing immune efficacy does not rely on high-intensity induction alone but rather on rhythm-based modulation of immune remodeling mechanisms, thereby achieving a dynamic balance between therapeutic potency and safety. This insight provides a new theoretical basis for optimizing immune strategies in ferroptosis-based therapies.

#### 6.2.3 Tissue-specific targeting

To enhance the selectivity of ferroptosis inducers in GC treatment and reduce systemic toxicity, tissue-specific promoter-driven systems and antibody-drug conjugates (ADCs) have attracted increasing attention as precision delivery strategies. HER2 is a common therapeutic target in GC, and HER2-positive tumors are responsive to antibody-based therapies such as Trastuzumab. Studies have explored conjugating ferroptosis inducers, such as RSL3 prodrugs or GPX4-targeting siRNA, to HER2 antibodies or their fragments (e.g., scFv), to construct siRNA–antibody fusion nanosystems capable of recognizing and binding to the surface of HER2-positive cells, thereby triggering endocytosis and targeted intracellular drug release. A study synthesized a gold nanoparticle-based dual-targeting platform co-loaded with HER2-siRNA and doxorubicin, which exhibited potent antiproliferative and ferroptosis-inducing effects in a HER2-positive breast cancer model, and achieved tumor-specific activation via a pH-responsive release system (151). Although this platform is currently applied in breast cancer, its structural versatility and the widespread expression of the HER2 target in GC suggest that it holds strong potential for translational application in GC therapy.

These innovative strategies collectively reflect the ongoing effort to enhance precision and safety in ferroptosis-based GC therapy. However, they also highlight a deeper, systemic challenge: ferroptosis is not a static or isolated event but a dynamic biological process intricately coupled with metabolism, redox homeostasis, and immune surveillance. Therefore, solely optimizing delivery or targeting may be insufficient to fully resolve off-target toxicity and variability in therapeutic outcomes. Tumor heterogeneity, spatiotemporal dynamics of the microenvironment, and adaptive resistance mechanisms further complicate precise ferroptosis induction. A deeper understanding of the interactions between ferroptosis, immune components, and stromal elements will be essential to provide a theoretical basis for preventing potential side effects. Ultimately, the successful transition of ferroptosis from a conceptual promise to a clinically viable therapy will require not only technological innovation but also a systems-level shift in precision oncology paradigms.

## 7 Discussion

Ferroptosis, an emerging form of regulated cell death, has garnered increasing attention in the field of cancer therapy, particularly in the context of GC, where it has demonstrated considerable therapeutic potential. Ferroptosis acts through multiple mechanisms—including iron metabolism imbalance, lipid peroxidation, and oxidative stress—to directly disrupt the survival microenvironment of tumor cells, and exerts a significant impact on the initiation, progression, and drug resistance mechanisms of GC. Recent studies have shown that ferroptosis not only inhibits the proliferation of GC cells but also promotes tumor cell death, and holds promising potential in limiting metastasis and enhancing the efficacy of chemotherapy and targeted therapies.

**Figure 6:**
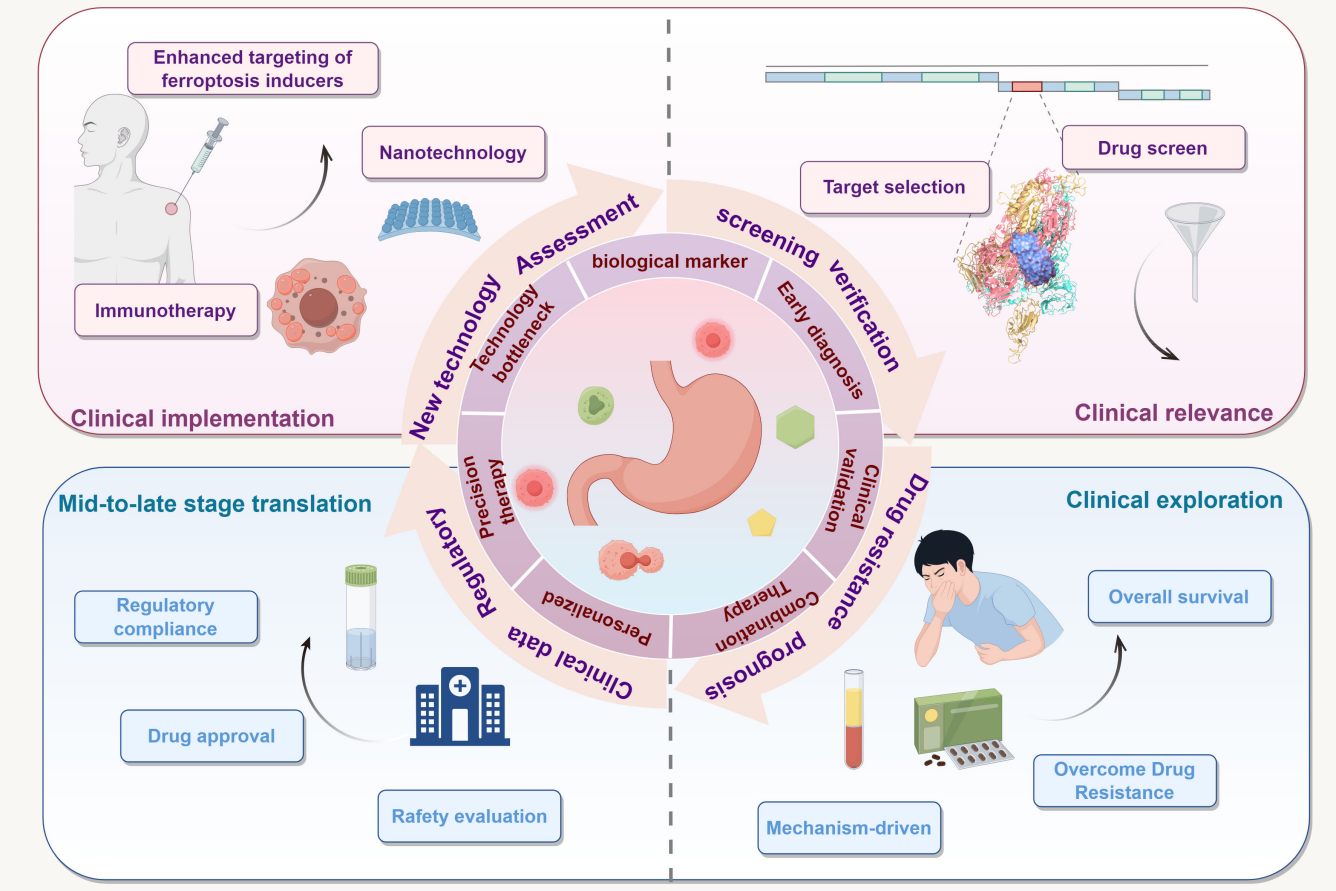
This figure illustrates the key translational pathway of ferroptosis from basic research to clinical application in the treatment of GC. The upper right quadrant represents the stage of target identification and drug screening, focusing on mechanistic exploration and clinical relevance validation. The lower right quadrant corresponds to the clinical exploration stage, encompassing resistance overcoming strategies, mechanism-driven approaches, and evaluation of survival benefits. The lower left quadrant presents the key tasks of the late-stage translational phase, such as drug approval, safety evaluation, and regulatory compliance. The upper left quadrant illustrates the clinical implementation stage, highlighting the potential application of ferroptosis inducers in combination with immunotherapy and nanotechnology in patients. At the center of the figure, a circular translational loop is constructed around GC cell biomarkers, forming a closed-loop path from "early diagnosis—clinical validation—combination therapy—precision therapy," which illustrates the multidimensional integrative mechanism of ferroptosis therapy from laboratory research to clinical practice.

Although several studies have highlighted the potential value of ferroptosis in the treatment of GC, significant challenges remain. The induction mechanisms of ferroptosis are complex, and different GC subtypes may exhibit heterogeneous responses to ferroptosis, necessitating greater emphasis on personalized therapeutic strategies in clinical translation. In addition, the selectivity and safety of ferroptosis inducers require further optimization to minimize their toxicity to normal cells. Future research should further investigate the role of ferroptosis across different stages and subtypes of GC, with a particular focus on elucidating its mechanisms, especially its interactions with the tumor microenvironment, immune evasion, and drug resistance. The following figure illustrates the key translational pathway of ferroptosis from basic research to clinical application in GC therapy, encompassing multiple stages, including target identification, biomarker validation, development of combination therapy strategies, and regulatory compliance, highlighting the multidimensional integration from mechanistic investigation to clinical implementation in this field (**Figure 6**).

Incorporating the concepts of precision medicine and personalized therapy, ferroptosis-based treatment holds promise for tailoring interventions to patients’ molecular profiles, thereby enhancing therapeutic efficacy and minimizing adverse effects. Overall, ferroptosis remains in the early stages of investigation in GC therapy. Despite existing challenges, ongoing advancements in molecular biology, drug development, and clinical translation continue to fuel its potential as a novel therapeutic strategy, offering improved prognosis and quality of life for patients.
